# Transplantation of bone marrow mesenchymal stromal cells attenuates liver fibrosis in mice by regulating macrophage subtypes

**DOI:** 10.1186/s13287-018-1122-8

**Published:** 2019-01-11

**Authors:** Xiao-Yu Luo, Xiang-Jun Meng, Da-Chun Cao, Wei Wang, Kun Zhou, Lei Li, Mei Guo, Ping Wang

**Affiliations:** 1 Department of Gastroenterology, Shanghai Ninth People’s Hospital, Shanghai Jiao Tong University School of Medicine, Center for Specialty Strategy Research of Shanghai Jiao Tong University China Hospital Development Institute, No 639, Zhizaoju Road, Huangpu District, Shanghai, 200011 China; 2 Department of Pathology, Affiliated Obstetrics and Gynecology Hospital of Nanjing Medical University, Nanjing Maternity and Child Health Care Hospital, Nanjing, Jiangsu province China; 3 Department of Pathology, Traditional Chinese Medicine Hospital of Kunshan, Kunshan, Jiangsu province China

**Keywords:** Mesenchymal stromal cell, Macrophage, Liver fibrosis, Matrix metalloproteinase 13, Hepatic stellate cell

## Abstract

**Background:**

Liver fibrosis is a key phase that will progress to further injuries such as liver cirrhosis or carcinoma. This study aimed to investigate whether transplantation of bone marrow mesenchymal stromal cells (BM-MSCs) can attenuate liver fibrosis in mice and the underlying mechanisms based on the regulation of macrophage subtypes.

**Methods:**

A liver fibrosis model was induced by intraperitoneal (i.p.) injection of CCl4 twice per week for 70 days, and BM-MSCs were intravenously transplanted twice on the 60th and 70th days. Immunohistology and gene expression of liver fibrosis and macrophage subtypes were analyzed. Mouse RAW264.7 cells and JS1 cells (hepatic stellate cell strain) were also used to explore the underlying mechanisms of the effects of BM-MSCs on liver fibrosis.

**Results:**

After transplantation of BM-MSCs, F4/80^+^CD206^+^-activated M2 macrophages and matrix metalloproteinase 13 (MMP 13) expression were significantly increased while F4/80^+^iNOS^+^-activated M1 macrophages were inhibited in liver tissue. Gene expression of IL-10 was elevated while IL12b, IFN-γ, TNF-α, and IL-6 gene expression were decreased. ΤGF-β1 and collagen-1 secretions were reduced while caspase-3 was increased in JS1 cells treated with BM-MSC-conditioned media. BM-MSCs effectively suppressed the expression of α-SMA, Sirius red, and collagen-1 in the liver, which are positively correlated with fibrosis and induced by CCl4 injection.

**Conclusions:**

Taken together, we have provided the first demonstration that BM-MSC transplantation can promote the activation of M2 macrophages expressing MMP13 and inhibition of M1 macrophages to further inhibit hepatic stellate cells (HSCs), which play synergistic roles in attenuating liver fibrosis.

## Background

Epidemiological analysis has revealed that liver fibrosis/cirrhosis is a severe health problem worldwide that accounts for substantial morbidity and mortality. Of the 1.4 million liver disease deaths each year, 55% are attributed to liver cirrhosis [1–4]. Liver fibrosis is a key period in the development of almost any liver disease that involves gradual destruction and will progress to liver cirrhosis or carcinoma. There are few effective treatments for curing liver fibrosis/cirrhosis and carcinoma, and liver transplantation remains the only option, which is restricted by a lack of donor organs and lifelong immunological rejection.

Mesenchymal stromal cells (MSCs) are currently attracting great attention from researchers because they are associated with fewer ethical concerns than embryonic stem cells; on the other hand, they are poor stimulators of the allogeneic T cell response in vitro and do not trigger a strong host inflammatory response in vivo [5, 6] because they only express low levels of type I HLA and do not express type II HLA and the costimulatory molecules CD40, CD80, and CD86 [5]. Recent studies have demonstrated that MSCs can be transplanted into baboons or even humans with beneficial effects and without immunological rejection, as well in most animals [7, 8].

Among various types of MSCs, bone marrow mesenchymal stromal cells (BM-MSCs) are now preferred not only due to their easy isolation and high expandability but also for their thoroughly characterized phenotypic expression, cytokine secretion, and paracrine activity [9]. Since Friedenstein et al. first described BM-MSCs [10], many properties of BM-MSCs have been reported, such as connecting different tissues, secreting various growth factors, anti-inflammation, and immunoregulation. BM-MSCs have been shown to play an anti-fibrosis role in animal models and in several human clinical trials [11, 12]. There are different perspectives about the anti-fibrosis mechanisms of BM-MSCs, and the latest research indicates that BM-MSCs reduce liver fibrosis via immunosuppressive and anti-inflammatory activities, such as inhibitory efforts on natural killers (NK) cells, dendritic cells, and Th1 cell proliferation and activation of M2 macrophages and Th2 cells [13]. Due to the abundance of innate immune cells in the liver, the polarization of macrophages after BM-MSC transplantation attracted our interest.

Macrophages include different subtypes, mainly M1 and M2 macrophages, according to their different surface markers, gene expression profiles, and activated effects [14, 15]. There have been few studies of the influence of macrophage subtypes on the liver fibrosis process. Initially, studies reported that M2 macrophages stimulated the development of liver fibrosis while M1 macrophages suppressed fibrosis, but Pesce et al. subsequently demonstrated that activated M2 macrophages inhibited fibrosis [16, 17]. Recently, a study reported that M1 macrophages accelerate the process of liver fibrosis [18]. And previous studies have shown that MMPs are essential for fibrinolysis, and MMP13 in particular, as the major interstitial collagenase in rodents, plays a crucial role in the resolution and cleavage of fibrous collagen [19–22]. However, no study has demonstrated relationships or mechanisms linking the transplantation of BM-MSCs and macrophage polarization with the expression of associated matrix metalloproteinases (MMPs) in a liver fibrosis model. Consequently, we examined the effect of the administration of BM-MSCs on liver fibrosis in mice and investigated the impact of BM-MSC transplantation on the regulation of macrophage subtypes and MMPs expression to determine the therapeutic potential of BM-MSCs in liver fibrosis.

## Material and methods

### Animal models

All animals received humane care, and all methods were carried out in accordance with the *Guide for the Care and Use of Laboratory Animals*. The experiments were approved by the Committee on the Ethics of Animal Experiments of Shanghai Jiao Tong University. Ten-week-old male C57BL/6J mice weighing 25–27 g were housed four per cage in temperature- and light-controlled chambers. There are a variety of experimental liver fibrosis models, but the CCl4-induced model appears to be the most classic and widely applied [23, 24]. In this study, liver fibrosis was induced by i.p. injection of CCl4 dissolved in olive oil at a volume ratio of 1:1 at a dose of 0.1 ml/mouse twice per week for 70 days. The animals were randomized into three groups as follows: (1) normal control group (*n* = 10)—treated with i.p. injection of saline twice per week for 70 days; (2) fibrosis group (*n* = 10)—treated with i.p. injection of CCl4 twice per week for 70 days; and (3) fibrosis+MSC group (*n* = 12)—treated with CCl4 twice per week for 70 days and treated with an injection of BM-MSCs via the tail vein at a dose of 5 × 10^5^ on the 60th day and 70th day. Animals were sacrificed on the 80th day, and each liver was excised and divided into several parts for hematoxylin-eosin (HE) staining, immunohistochemical staining, immunofluorescence staining, and RNA extraction. In addition, five mice were treated with CCl4 followed by transplantation of GFP-positive BM-MSCs to assess the migration of the transplanted cells.

### Isolation, expansion, and characterization of BM-MSCs

BM-MSCs were isolated and cultured as described in a previous study [25]. Briefly, after the donor mice were sacrificed, the cleaned bones of the tibia and femur were stored in DMEM (Gibco, Carlsbad, CA, USA) supplemented with penicillin/streptomycin on ice. The bone marrow was extracted by inserting a 27-gauge needle attached to a 10-ml syringe containing DMEM with strong flushing to remove the growth plates of the bones. The cell suspension was filtered through a 70-μm filter mesh and then cultured in a 60-mm culture dish in 1 ml of complete medium at a density of 25 × 10^6^/ml. The plate was incubated at 37 °C with 5% CO_2_ in a humidified chamber. After 6 h, nonadherent cells were removed by replacing the medium with fresh complete medium. After an additional 6 h of culture, the medium was replaced by 1.5 ml of fresh complete medium. Afterwards, the medium was changed every 8 h for up to 3 days of initial culture. Then, adherent cells were washed with PBS, and the medium was replaced with 6 ml of fresh medium every 3 to 4 days. From the third day, spindle-shaped cells appeared and expanded to become increasingly confluent. At the third week, highly purified BM-MSCs were obtained. And the cells from P5–7 were used for further experiments in this study.

The BM-MSCs were characterized using suitable markers by flow cytometric analysis. BM-MSCs were CD54^+^ CD90^+^ CD11^−^ in this experiment. The FACS analysis was performed using a CyAn ADP flow cytometer (Beckman Coulter). All data were analyzed by FlowJo software (TreeStar, Inc). Differentiation experiments were performed using a mesenchymal stromal cell adipogenic differentiation kit and osteogenic differentiation kit (R&D Systems Minneapolis, MN, USA) to establish the reliability of the BM-MSCs.

### Histopathological and immunohistochemical examination

Liver tissue samples were stored in 10% formalin solution. Paraffin blocks were prepared as 4-μm cross sections, and HE staining and Sirius red staining were performed. Formalin-fixed and paraffin-embedded sections of the livers were also used in the immunohistochemical examination, and α-smooth muscle actin (α-SMA) staining was applied to show the activation of HSCs (dilution 1:100; Dako Japan, Tokyo, Japan). Collagen-1 staining was conducted to show the fibrous collagen of liver fibrosis (dilution 1:100; Abcam, Cambridge, MA, USA). The fibrotic areas were observed in three sections per mouse.

### Immunofluorescence staining

Liver tissue was immediately obtained when the mice were sacrificed and subsequently dehydrated in 30% sucrose PBS solution, embedded in Tissue-Tek OCT compound (Sakura Finetek USA, Inc., Torrance, CA, USA), and snap-frozen in dry ice. Frozen sections with a thickness of 6 μm were fixed in 4% paraformaldehyde, blocked with 5% goat serum, and incubated at 4 °C overnight with primary antibodies against F4/80 (marker of mouse monocytes/macrophages), iNOS (marker of mouse M1 macrophages), CD206 (marker of mouse M2 macrophages), and MMP13. All of the above antibodies (Abcam, Cambridge, MA, USA) were diluted 1:100. The frozen sections were incubated with appropriate fluorescein-conjugated secondary antibodies for 2 h at room temperature. The fluorescence was examined and photographed using a Lecia fluorescence microscope.

### RNA preparation and quantitative reverse transcriptase polymerase chain reaction

Total RNA was extracted from frozen liver tissue using Isogen (Nippon Gene, Tokyo, Japan). Each 800-ng RNA sample was reverse-transcribed to cDNA using oligo (dT) primers and SuperScript reverse transcriptase (Invitrogen, Life Technologies Japan) according to the manufacturer’s protocol. The target-specific primers were designed as listed in Table 1. Quantitative RT-PCR was performed using a TaqMan system on an Applied Biosystems PRISM7700 device (ABI Japan, Co., Ltd., Tokyo, Japan) with 0.9 mM each primer in a final reaction volume of 25 μl of Premix Ex TaqTM (Takara Bio Inc., Shiga, Japan). The PCR cycling conditions were as follows: 50 °C for 2 min, 95 °C for 15 min, and 50 cycles of 95 °C for 30 s, 60 °C for 1 min, and 25 °C for 2 min. The data were expressed as the comparative cycle threshold (*C*_*t*_) values. The normalized *C*_*t*_ value of each gene was obtained by subtracting the *C*_*t*_ value of 18 s rRNA.

**Table 1:** The primers used in this study

Genes	Forward (5′-3′)	Reverse (5′-3′)
IL-6	CTGCAAGTGCATCATCGTTGT	TGTCTATACCACTTCACAAGTCGGA
TNF-α	TGTCTACTGAACTTCGGGTGAT	AACTGATGAGAGGGAGGCCAT
IFN-γ	CAAGGCGAAAAAGGATGCA	CGGATGAGCTCATTGAATGCT
IL-10	GGGTGAGAAGCTGAAGACCCT	TCACCTGCTCCACTGCCTT
TGF-β1	AGGTCACCCGCGTGCTAA	GCTTCCCGAATGTCTGACGTA
α-SMA	CTGACAGAGGCACCACTGAA	CATCTCCAGAGTCCAGCACA
IL-12b	CTCAGAAGCTAACCATCTCCTGG	CACAGGTGAGGTTCACTGTTTC
MMP13	TTATGGTCCAGGCGATGAAG	AGGCGCCAGAAGAATCTGTC
Collagen-1	GAGCGGAGAGTACTGGATCG	TACTCGAACGGGAATCCATC
Collagen-4	GGTATTCAGGGAGACCGTGG	ACCCTTGTGCACCCCTAGAT
18s	ATGAGTCCACTTTA	CTTTAATATACGCT
	AATCCTTTAACGA	ATTGGAGCTGGAA

### M1 macrophage polarization and co-culture assay

Murine RAW264.7 cells and JS1 cells (hepatic stellate cell strain) obtained from the Cell Bank of the Chinese Academy of Sciences (Shanghai, China) were used for further experiments in this study which were from P4–5. The cells were cultured in DMEM supplemented with 10% FBS (Gibco, USA), 100 U/ml penicillin, and 100 μg/ml streptomycin at 37 °C in a humidified 5% CO_2_ atmosphere. For experiments, the RAW264.7 cells (seeded at 3 × 10^5^/ml) were stimulated with 100 ng/ml LPS for 6 h as described previously [26] to yield M1 macrophage polarization.

JS1 cells and LPS-stimulated M1 macrophages from RAW264.7 cells were co-cultured in two chambers separated by a semipermeable membrane with a pore size of 1 μm to prevent contact between the cells. JS1 cells were cultured in the upper insert of the chamber, while M1 macrophages were cultured in the lower chamber. In addition, BM-MSC-conditioned media was added to the chamber containing M1 macrophages for stimulation, and the impact of the influence of the BM-MSCs on M1 macrophages on the activation or apoptosis of JS1 cells was observed. After 48 h of co-culture, the upper inserts were removed, and the levels of ΤGF-β1 and collagen-1 in the supernatant of the JS1 cells were measured by ELISA (R&D Systems Minneapolis, MN, USA). Caspase-3 was measured in lysed JS1 cells. The levels of ΤGF-β1 and collagen-1 were also measured in the supernatants of M1 macrophages and JS1 cells when they were cultured alone with or without BM-MSC-conditioned media.

### Measurement of caspase-3 enzyme activity in JS1 cells

Caspase-3 enzyme activity was measured using a Caspase-3 Activity Assay kit (Beyotime, Shanghai, China) according to the manufacturer’s instructions. Briefly, JS1 cells were harvested after 48 h of culture alone or co-culture with LPS-stimulated M1 macrophages from RAW264.7 cells that were treated with or without BM-MCS-conditioned media. The harvested cells were then lysed in cold lysis buffer and centrifuged at 15,000*g* for 5 min. The supernatant was transferred to ice-cold fresh tubes for immediate assay. The assay was based on the spectrophotometric detection of chromophore p-nitroaniline (*p*-NA) after cleavage from the labeled substrate DEVD-*p*-NA. The *p*-NA fluorescence emission was quantified at 405 nm, and finally, the caspase-3 activity was determined by comparison of the absorbance of *p*-NA from the treated sample with that of the control.

### Statistical analysis

The results were presented as the means ± SE, and the data were analyzed using the statistical software package SPSS 12.0 (SPSS Inc., Chicago, IL, USA). The groups were compared by one-way ANOVA, followed by Fisher’s protected least significance difference test or the Mann-Whitney *U* test. Values of *p <* 0.05 were considered to be statistically significant.

## Results

### Characterization of BM-MSCs

**Fig. 1:**
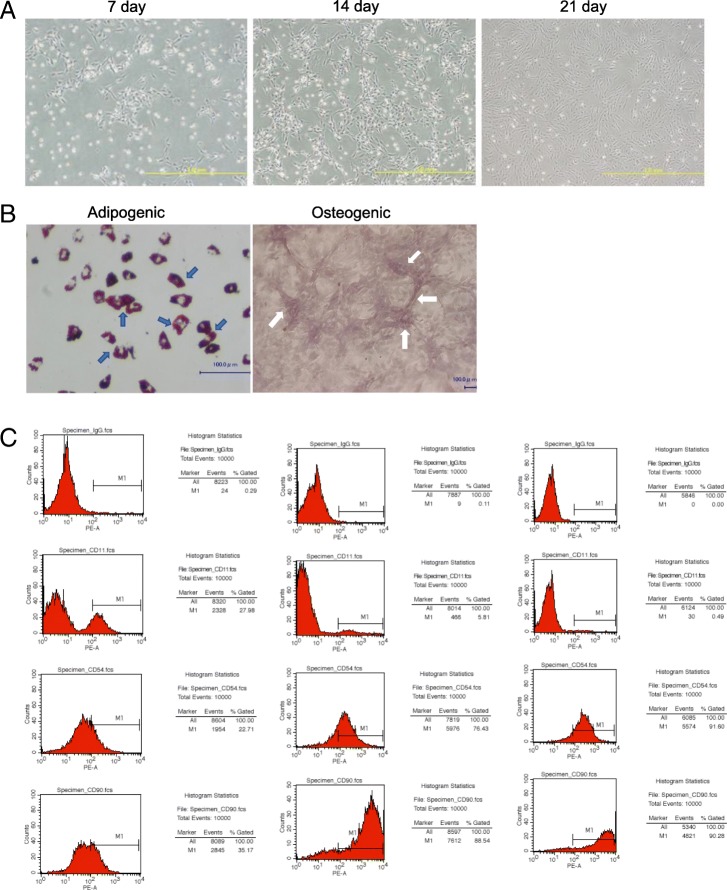
Morphological, immunophenotypic, and differentiation analysis of BM-MSCs. **a** Morphological pictures of BM-MSCs after 7 days, 14 days, and 21 days of culture. The scale bars represent 1.0 mm. **b** BM-MSCs differentiated into adipocytes (blue arrows) and osteoblasts (white arrows). The scale bars represent 100 μm. **c** Flow cytometric analysis of BM-MSCs (CD90^+^ CD54^+^ CD11^−^) in different culture time

BM-MSCs were isolated and cultured following our above protocol. The BM-MSCs reached 25–35% confluence after 7 days and 75–85% confluence after 14 days. After 21 days of culture, the essentially uniform spindle-shaped BM-MSCs reached greater than 92% confluence as assessed by phase-contrast microscopy (Fig. 1a), and more than 70% of cells had a colony-formation capacity, consistent with a previous report [25]. Furthermore, we verified the purification process and reliability of the BM-MSCs by flow cytometric analysis and differentiation experiments. CD11^+^mononuclear macrophages and granulocytes gradually decreased while CD90^+^ CD54^+^ CD11^−^ BM-MSCs gradually increased from the first week to third week (Fig. 1c). The BM-MSCs exhibited little contamination by hematopoietic or other cell lineages after 3 weeks of culture (Fig. 1a, c). Differentiation experiments showed that the BM-MSCs successfully differentiated into adipocytes and osteoblasts after 3 weeks of induction, based on visualization of oil droplets in the cultured cells by positive Oil Red O staining and calcium-containing precipitates by staining with 2% Alizarin red adjusted to a pH of 4.4 with ammonium hydroxide (Fig. 1b).

### BM-MSCs migrated to the injured livers and attenuated the loss of body weight and liver injury

**Fig. 2:**
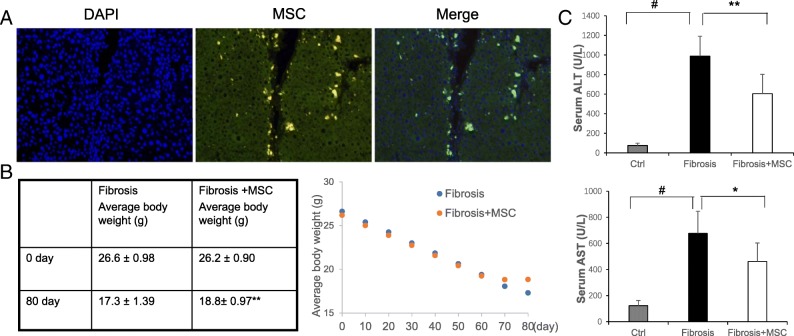
Transplanted BM-MSCs migrated to the injured liver and alleviated the loss of body weight and liver injury. **a** Migration of GFP-positive BM-MSCs into the liver after 24 h (× 200 magnification). **b** The body weight loss in the fibrosis group and fibrosis+MSC group. **c** The ALT and AST levels in each group. (BM-MSCs were from P5–7, means ± SE; ^#^*p* < 0.01 vs. the normal control group, **p < 0.01 vs. the fibrosis group, *p < 0.05 vs. the fibrosis group)

To assess the migration of transplanted BM-MSCs, we injected 5 × 10^5^ BM-MSCs from GFP mice in each liver-injured recipient via the tail vein. Abundant GFP-positive BM-MSCs were detected in the recipient mice after transplantation (Fig. 2a). The number of GFP-positive cells reached a maximum between 12 and 36 h after transplantation.

From the beginning of the experiment until the 60th day, the average body weight was not significantly different between the fibrosis group and fibrosis+MSC group. However, after injection of BM-MSCs twice on the 60th day and 70th day, the loss of body weight slowed in the fibrosis+MSC group. At the end point (the 80th day) of the experiment, the weight decreased by 9.3 g on average in the fibrosis group and 7.4 g on average in the fibrosis+MSC group, corresponding to approximately 35% and 28.2%, respectively, of their initial body weights (Fig. 2b). In addition, the injection of CCl4 twice per week for 70 days resulted in 13- and 5.5-fold increases in serum alanine aminotransferase (ALT) and aspartate aminotransferase (AST) levels, respectively, compared with normal mice; however, transplantation of BM-MSCs effectively inhibited the increase in serum aminotransferase (Fig. 2c).

### Transplantation of BM-MSCs suppressed liver fibrosis

**Fig. 3:**
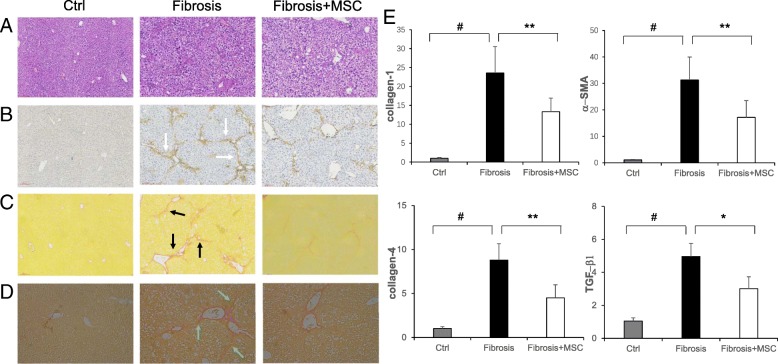
**a** Histological HE staining. **b** α-SMA staining; white arrows indicate α-SMA-positive cells. **c** Sirius red staining; black arrows indicate Sirius red-positive areas. (**a**–**c**, × 100 magnification). **d** Collagen-1 staining; green arrows indicate collagen-1-positive areas (× 200 magnification). **e** The expression of liver fibrosis-related genes in each group. (means ± SE; ^#^*p* < 0.01 vs. the normal control group, **p < 0.01 vs. the fibrosis group, *p < 0.05 vs. the fibrosis group)

Mice that did not receive CCl4 injection displayed normal histology, while mice that received CCl4 injection twice per week for 70 days developed obvious hepatic fibrosis. By contrast, mice that received CCl4 and BM-MSC injections displayed an apparent decrease in fibrosis (Fig. 3a). Furthermore, we detected α-SMA, Sirius red, and collagen-1 staining, which represent the extent of liver fibrosis. Consistent with the HE staining, α-SMA staining showed numerous positive cells located around the central vein areas and infiltrated into the middle part of the lobules in the fibrosis group. However, α-SMA-positive areas were significantly reduced in the fibrosis+MSC group (Fig. 3b). Sirius red staining showed marked perisinusoidal collagen deposition starting from the central district and extending into the hepatic lobules, which occupied 25% of the liver area in the fibrosis group, while this amount of collagen was decreased to 12% of the liver area in the fibrosis+MSC group (Fig. 3c). The collagen-1 staining results were similar to the α-SMA and Sirius red staining results (Fig. 3d).

We also measured fibrosis-related gene expression levels in liver tissue, including TGF-β1,α-SMA, collagen-1, and collagen-4. TGF-β1 was largely secreted by activated HSCs, which will accelerate the formation of liver fibrosis, and α-SMA was expressed mainly by myofibroblasts derived from activated HSCs. The mRNA expression levels of the above four genes were low in normal mice and were obviously increased by CCl4 administration. Notably, the mRNA expression levels of these genes were dramatically decreased by BM-MSC transplantation (Fig. 3e).

### Transplantation of BM-MSCs increased the M2/M1 macrophage ratio

**Fig. 4:**
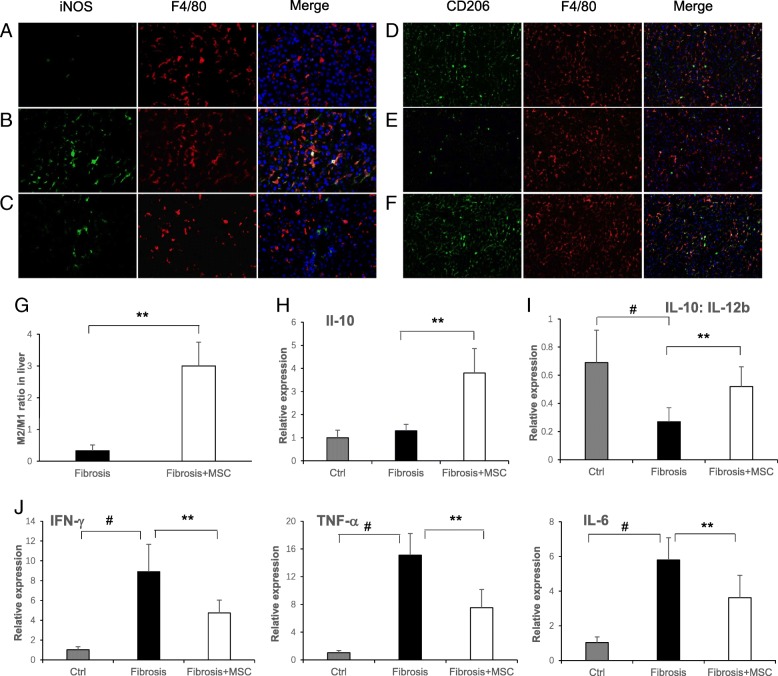
Transplantation of BM-MSCs induced activation of M2 macrophages and inhibition of M1 macrophages. **a–f** Immunofluorescence analysis of M1 and M2 macrophages (**a**, **d** normal control; **b**, **e** fibrosis; **c**, **f** fibrosis+MSC). **g** The M2/M1 ratio was determined in six randomly selected high-power fields. **h** M2 macrophage-related IL-10 mRNA expression. **i** The IL-10/IL-12b ratio. **j** The relative mRNA expression of IFN-γ, TNF-α, and IL-6 in the liver. (BM-MSCs were from P5–7, means ± SE; ^#^*p* < 0.01 vs. the normal control group, **p < 0.01 vs. the fibrosis group)

Immunofluorescence staining of M1 and M2 macrophage markers revealed some interesting changes in macrophage subtypes. F4/80^+^iNOS^+^ cells represented activated M1 macrophages, while F4/80^+^CD206^+^ cells represented activated M2 macrophages. M1 macrophages dramatically increased in fibrotic livers induced by CCl4 but decreased significantly after BM-MSC transplantation (Fig. 4b, c). However, M2 macrophages displayed an obvious decrease in the fibrosis group but a dramatic increase in the fibrosis+MSC group (Fig. 4e, f). Thus, transplantation of BM-MSCs effectively increased the M2/M1 macrophage ratio in the liver (Fig. 4g).

To further confirm the effect of BM-MSCs on regulating macrophage subtypes, we detected M1 and M2 macrophage-related cytokines. IL12b is derived from M1 macrophages, and IL-10 is mainly derived from M2 macrophages [27]. We found that the mRNA level of IL12b was significantly increased in the fibrotic livers but decreased after transplantation of BM-MSCs two times. However, IL-10 mRNA showed a different change trend, with a marked increase in the fibrosis+MSC group compared with the fibrosis group (Fig. 4h). In addition, the IL-10/IL12b ratio was decreased in the fibrotic liver induced by CCl4 but increased by the administration of BM-MSCs (Fig. 4i). We also measured the expression of M1 macrophage-related inflammatory factors including IFN-γ, TNF-α, and IL-6 in liver tissue, which simultaneously increased in the fibrosis group but obviously decreased in the fibrosis+MSC group (Fig. 4j).

### BM-MSC transplantation increased the expression of MMP13 by activated M2 macrophages

**Fig. 5:**
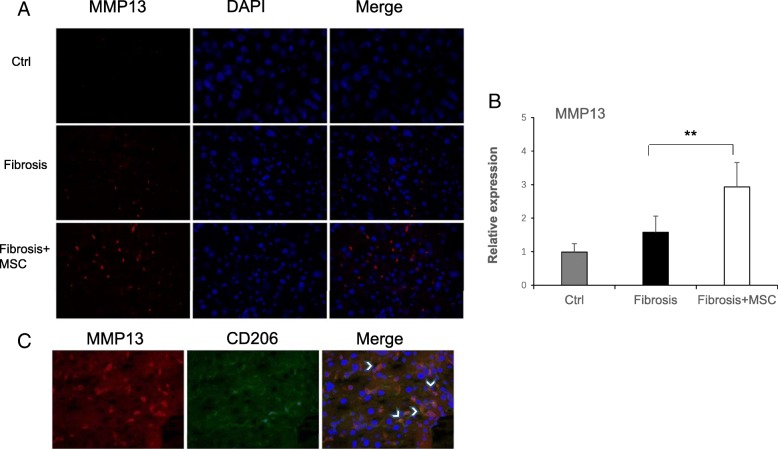
**a** Immunofluorescence staining of MMP13 in the liver. **b** The relative mRNA expression of MMP13 in each group (means ± SE; **p < 0.01 vs. the fibrosis group). **c** Double immunofluorescence staining; the white arrowheads indicate MMP13-positive cells where CD206 was co-immunolocalized

The components of fibrous collagen are mainly type I collagen in liver fibrosis, which is largely degraded by MMP1 in humans. Rodent MMP1 has not been identified, but studies have shown that MMP13 plays an equivalent role in rodents [19]. Immunofluorescence staining of MMP13 showed that MMP13 expression was increased in the fibrosis+MSC group compared with the fibrosis group (Fig. 5a). In addition, the mRNA expression level of MMP13 was consistent with the results of immunofluorescence staining (Fig. 5b). Moreover, we performed double immunofluorescence staining of MMP13 and CD206 and found that the positive areas of MMP13 expression and CD206 expression overlapped by more than 90% (Fig. 5c). These results verified our speculation that the increased MMP13 expression was derived from activated and proliferative M2 macrophages.

### Effect of BM-MSC-conditioned media on HSCs via M1 macrophages

**Fig. 6:**
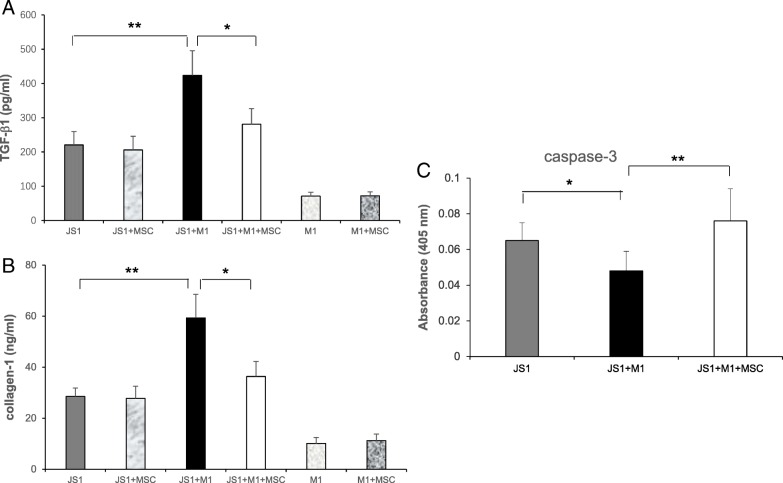
BM-MSC-conditioned media inhibited HSCs via suppressing M1 macrophages. BM-MSC-conditioned media decreased ΤGF-β1 production (**a**) and collagen-1 production (**b**) in JS1 cells and induced an increase in caspase-3 production in JS1 cells (**c**). (BM-MSCs were from P5–7, JS1 cells and RAW264.7 cells were from P4–5, means ± SE; **p < 0.01, *p < 0.05)

ΤGF-β1 and collagen-1 were detected by ELISA in the supernatants of six groups: JS1, JS1+MSC, M1, M1+MSC, JS1+M1, and JS1+M1+MSC. The results revealed that the concentrations of ΤGF-β1 and collagen-1 in JS1 cells cultured alone were increased by 3.1- and 2.8-fold, respectively, compared with M1 macrophages cultured alone. No obvious changes in concentration were detected after incubation with BM-MSC-conditioned media. However, the levels of ΤGF-β1 and collagen-1 were apparently increased in the JS1+M1 co-culture group compared with JS1 cells cultured alone. Furthermore, the addition of BM-MSC-conditioned media to M1 macrophages in the co-culture group ultimately reduced the levels of secreted ΤGF-β1 and collagen-1 in JS1 cells (Fig. 6a, b). We also detected the apoptosis of JS1 cells when cultured alone or co-cultured with M1 macrophages treated with or without BM-MSC-conditioned media. The results showed that caspase-3 was decreased in the co-culture compared with JS1 cells cultured alone. In addition, caspase-3 production in the co-culture was significantly increased after BM-MSC-conditioned media was added to the M1 macrophage culture (Fig. 6c).

## Discussion

Liver fibrosis, the key phase that may eventually progress to liver cirrhosis or hepatocellular carcinoma, is a complex and kinetic process involving various cell types and cytokines that result in the activation of hepatic stellate cells and accumulation of excessive extracellular matrix (ECM). However, there are no satisfactory treatments for liver fibrosis. New therapies such as stromal cell transplantation have shown improvements in liver biochemical parameters and histological evaluation, and further research on these therapies is urgently needed.

In our study, CCl4 induced a loss of body weight and increased ALT and AST levels, although some previous studies reported that body weight increased slightly in mice receiving CCl4 injections for 6 to 8 weeks compared to the initial body weight. Notably, the injection of CCl4 induced a dramatic decrease in body weight compared with the normal control in all of those studies, similar to our data. Importantly, BM-MSC transplantation attenuated the loss of body weight and inhibited the increase in aminotransferases in our present study (Fig. 2b, c). We attributed these changes to the induction of hepatocyte injury by CCl4 injection and the reduced synthesis of albumin in the liver, which further induced the loss of body weight; transplantation of BM-MSCs rectified this hepatocyte injury and restored liver function to a certain extent.

As expected, CCl4 injection promoted obvious liver fibrosis, which was effectively attenuated by BM-MSC transplantation in our present study. Immunohistochemical staining of α-SMA showed that CCl4 induced a notable increase in myofibroblasts, and this increase was reduced significantly by transplantation of BM-MSCs (Fig. 3b). α-SMA-positive cells were used to represent myofibroblasts, which are derived from activated HSCs [28], and it is universally accepted that activation of HSCs plays a key role in the process of liver fibrosis [29]. The change in positive areas of Sirius red and collagen-1 staining also demonstrated the effect of BM-MSCs in reducing liver fibrosis (Fig. 3c, d). The mRNA expression levels of TGF-β1, α-SMA, collagen-1, and collagen-4 further corroborated these results (Fig. 3e).

Notably, immunofluorescence staining of macrophage markers showed that CCl4 injection induced the proliferation of M1 macrophages, while BM-MSC transplantation induced the proliferation and activation of M2 macrophages and inhibition of M1 macrophages (Fig. 4b, c, e, f). M1 and M2 macrophage-related cytokines in liver tissue showed similar changes. Mice that received CCl4 followed by BM-MSC transplantation displayed a significant increase in IL-10 compared with mice that received CCl4 injection only, and the ratio of IL-10/IL12b was apparently increased by the administration of BM-MSCs (Fig. 4h, i). This result is also supported by a previous study that revealed that human amniotic epithelial cell transplantation induced markers of alternative macrophage activation [27].

Furthermore, our study not only demonstrated that transplantation of BM-MSCs alleviated liver fibrosis but also revealed that this alleviation effect was due to the elevated expression of MMP13 (Fig. 5a, b), which was consistent with previous studies reported MMPs especially MMP13 was the major interstitial collagenase in rodents, played a crucial role in degrading fibrous collagen [19–22]. Importantly, MMP13 was expressed by activated M2 macrophages in our present study (Fig. 5c), which accounted for the positive connection between the activation of M2 macrophages and attenuation of liver fibrosis. Hence, we concluded that transplantation of BM-MSCs attenuated liver fibrosis by activating M2 macrophages, which were able to express MMP13.

In addition, we detected the expression of inflammatory cytokines including IFN-γ, TNF-α, and IL-6, which simultaneously increased after CCl4 injection but decreased significantly after BM-MSC transplantation two times (Fig. 4j). The levels of the above inflammatory cytokines were closely associated with the change in M1 macrophages, demonstrating that these cytokines were mainly derived from M1 macrophages, consistent with results reported by Subramanian [30]. Our previous study provided apparent evidence of IFN-γ dependence of liver fibrosis [31]. Studies have also demonstrated that TNF-α and IL-6 play important roles in the development of liver fibrosis [32–34]. Our observations that CCl4 injection induced elevation of IFN-γ, TNF-α, and IL-6 and aggravation of liver fibrosis in this study are consistent with these previous conclusions. Taken together, these results indicated that the fibrosis-alleviating effect of BM-MSC transplantation was accompanied by a decrease in M1 macrophages and inhibition of the above relevant inflammatory cytokines. The inhibition of the activation of M1 macrophages, suppression of inflammatory cytokines, and decrease in HSC-related fibrous collagen after BM-MSC transplantation led us to hypothesize that the transplanted BM-MSCs probably inhibited a pathway from activation of M1 macrophages to activation of HSCs that normally would trigger the differentiation of α-SMA-positive myofibroblasts under stimulation. Hence, we further analyzed the influence of BM-MSCs on HSCs via M1 macrophages in vitro. The results showed that M1 macrophages induced the activation of HSCs; however, BM-MSCs were able to ultimately suppress the activation effect and accelerate the apoptosis of HSCs (Fig. 6).

## Conclusions

In conclusion, irritative factors such as CCl4 injection stimulate the proliferation of M1 macrophages, which further trigger HSC activation into α-SMA-positive myofibroblasts to accelerate the development of liver fibrosis by expressing TNF-α, IFN-γ, and IL-6. During the development of liver fibrosis, at least in the CCl4-induced liver fibrosis model, M2 macrophages were suppressed. However, transplantation of BM-MSCs effectively promoted the proliferation and activation of M2 macrophages expressing MMP13 and inhibited M1 macrophages to suppress the activation of HSCs, which together played synergistic roles in degrading liver fibrosis. Although research on the treatment of liver fibrosis by MSC transplantation has emerged in recent years, previous studies have usually focused on whether these stromal cells differentiate into hepatocyte-like cells to promote the regeneration of hepatic parenchymal cells and restore liver function [9, 35–37]. By contrast, the role of macrophages, as abundant innate immune cells in the liver, has been ignored. Our study is the first to demonstrate that the effects of BM-MSC transplantation on liver fibrosis are at least partially or even mainly based on their modulatory effect, especially via regulating macrophage subtypes. The change in macrophages plays a central role because it orchestrates cross-talk among different cell types, cytokines, and proteases to ultimately attenuate liver fibrosis.

## Abbreviations

BM-MSCs: Bone marrow mesenchymal stromal cells
CCl4: Carbon tetrachloride
CD: Clusters of differentiation
DMEM: Dulbecco’s modified Eagle media
GFP: Green fluorescent protein
HLA: Human leucocyte antigen
HSC: Hepatic stellate cell
IFN: Interferon
IL: Interleukin
i.p.: Intraperitoneal
MMP: Matrix metalloprotease
PBS: Phosphate buffer saline
SMA: Smooth muscle actin
TGF: Transforming growth factor
Th cell: Helper T cell
TNF: Tumor necrosis factor
